# Role of HE4 in evaluation of adnexal masses and its comparison with CA125, ROMA and RMI in premenopausal women

**DOI:** 10.4314/ahs.v24i4.16

**Published:** 2024-12

**Authors:** Mini Sharma, Neeraj Kumar, Subhas Saha, Vanita Suri, GR Prasad, Radhika Srinivasan, Arnab Pal

**Affiliations:** 1 Post Graduate Institute of Medical Education and Research, OBG; 2 Dr Rajendra Prasad Government Medical College; 3 Postgraduate Institute of Medical Education and Research, Chandigarh, Cytology and Gynecological Pathology; 4 Post Graduate Institute of Medical Education and Research, Biochemistry

**Keywords:** Adnexal mass, CA125, HE4, Ovarian neoplasms, Risk of malignancy index (RMI), Risk of malignancy algorithm (ROMA)

## Abstract

**Background:**

Ovarian cancer is the seventh most common cancer in women and is ranked third among gynaecological malignancies after cervical and uterine cancers. Prospective studies have failed to establish a definite screening programme based on tumour markers or ultrasonography.

**Objective:**

To evaluate potential role of Human Epididymis protein 4 (HE4) as a biomarker for diagnosis of various ovarian malignancies in premenopausal age group, either alone or as a part of diagnostic algorithm like Risk of Malignancy Algorithm (ROMA) and to analyse if it has any advantage over Cancer Antigen 125 (CA125) or Risk of Malignancy Index (RMI).

**Methods:**

It was an observational cross-sectional study which included 100 premenopausal women having ovarian mass and underwent surgery. The diagnostic performances of CA125, HE4, ROMA score and RMI for ovarian cancer were evaluated.

**Results:**

Postoperative histopathology confirmed 30% (n=30) women to have malignant ovarian tumors. According to receiver operating characteristic (ROC) analysis; area under curve (AUC) was maximum for ROMA (0.791) followed by HE4 (0.784), RMI (0.750) and CA125 (0.715).

**Conclusion:**

HE4 is not superior to CA125 but, it can be used in series or as part of diagnostic algorithm (ROMA) along with CA125 to get higher diagnostic accuracy for premenopausal women.

## Introduction

Ovarian cancer is the seventh most common cancer in women and is ranked third among gynaecological malignancies after cervical and uterine cancers 1. 5 year survival rates are much lower for ovarian cancer compared to other cancers that affect women because 70% of ovarian malignancies are diagnosed in advanced stage 2, 3. Prospective studies have failed testablish a definite screening programme based on tumour markers or ultrasonography 4, 5. Cytology based screening test cannot be developed for ovarian malignancy due to relative inaccessibility of ovaries as compared to other pelvic organs and its intraperitoneal location. Transabdominal or transvaginal Fine Needle Aspiration Cytology (FNAC) is described as a potential method but with obvious risk of rupturing an intact capsule and intraperitoneal spread of the malignant cells.

Cancer Antigen 125 (CA125) is the most frequently used biomarker for ovarian malignancy having established role in ovarian malignancy in postmenopausal women with high sensitivity and specificity 3. But in premenopausal women it has high false positive rate since it is falsely elevated in some benign ovarian conditions (which are more common in premenopausal age group) and diseases other than those that affect the ovary like first trimester of pregnancy, breast cancer, endometriosis, PID (Pelvic Inflammatory Disease) and other malignancies besides ovarian cancer (breast, colon, pancreatic, lung, gastric, liver cancer) 6.

The other commonly used modality transvaginal ultrasonography has shown remarkably high (>95%) sensitivity for the detection of early-stage ovarian cancer. But this test alone will lead to performance of as many as 10 to 15 laparotomies for each case of ovarian cancer detected 3. The other limiting feature of imaging is the subjective nature of sonography. Operator experience and variable reporting of morphologic features of an adnexal mass contribute to inconsistencies observed from centre to centre studies 7. Human Epididymis Protein 4 (HE4) is a novel tumor marker for epithelial ovarian cancer which is more specific for epithelial ovarian malignancies 8. Combined HE4 and CA125 had higher sensitivity than HE4 (73%) and CA125 (43%) alone in both premenopausal and postmenopausal women^9^. HE4 levels are increased in more than 50% of tumours that do not express CA125(9). HE4 is not elevated in benign ovarian conditions like in PID, dermoid and endometriosis(10). Diagnostic algorithm ROMA (Risk of Malignancy Algorithm) index value has been introduced which incorporate both HE4 and CA125 7. We evaluated the potential role of HE4 as a biomarker for diagnosis of various ovarian malignancies specifically in premenopausal age group, either alone or as a part of diagnostic algorithm like ROMA and analysed if it has any advantage over CA125 or RMI.

## Methods

This was an observational cross-sectional study approved by the Institutional Ethics Committee and was conducted between July 2016 and October 2018.

### Inclusion criteria

woman ≤ 50 years of age and had not attained menopause. Menopause was defined as woman over 40 years who had not experienced menses for at least one year. Already diagnosed with pelvic mass most likely of ovarian origin and were elected for surgery. Exclusion criteria Previous history of radiotherapy or chemotherapy. Non-consenting patients. Total of 100 patients could be recruited having age between 18 and 50 years (mean age 34.4 ± 8.71 years). Before surgery, blood samples of the patient were collected. The samples were centrifuged and serum was stored at –200C until used. Serum CA125 and HE4 levels were analysed by electro chemi luminiscence autoanalyzer COBAS E601 (Roche Diagnostics, Indianapolis, IN, USA) after proper calibration and quality controls using manufacturer's protocol. The cut off values for CA125 and HE4 were taken as 35 U/ml and 70 pM respectively.

RMI was calculated according to the criteria described by Jacobs et al 12. A score of one point each was assigned for the following ultrasound features suggestive of malignancy: presence of a multilocular cystic lesion, solid areas, bilateral lesions, ascites, and intra-abdominal metastases.

RMI = U x M x CA125,

where a total ultrasound score of 0 made U =0, a score of 1 made U =1, and a score of ≥2 made U =3; premenopausal status made M =1. The serum level of CA125 (U/ml) was applied directly to the calculation. The cut off RMI value for differentiating between benign versus malignant masses was taken as 200 (13). ROMA Index calculation was done as described by Moore et al^7^.

Premenopausal Predictive Index (PI) = 12.0+2.38xLogn (HE4)+ 0.0626xLogn(CA125)

ROMA cut off values for high-risk patients were taken as ≥13.1% for premenopausal women.

Statistical analysis was done using IBM SPSS STATISTICS (version 23.0) and Medcalc (version 18). Measurable data was tested for its normality about outcome using Kolmogorov Smirnov test. For normally distributed data, the group means were compared using Student's t-test. Whereas for skewed data, the distribution of the outcome over various parameters was compared using Mann Whitney test and Kruskal Wallis test. For statistical comparisons, a level of p<0.05 was accepted as statistically significant. Sensitivity, specificity, positive predictive value and negative predictive value for CA125, HE4, ROMA, and RMI were calculated.

## Results

The mean age of the cohort (n=100) was 34.4 ± 8.71 years. Mean age for benign, borderline and malignant diseases was found to be 33.8 ± 8.6 years, 33.78 ± 8.9 years and 35.88 ± 9.0 years respectively. Laparoscopy was performed for 36% (n=36) and laparotomy was performed for 64% (n=64). Laparotomy was performed in case malignancy was suspected or where laparoscopy was considered difficult due adhesions or previous surgery. 30% (n=30) women were confirmed to have malignant ovarian tumors on postoperative histopathology. Final histopathological outcomes of the 100 patients are shown in table 1.

**Table 1:** Histological Type and Distribution of Benign and Malignant Cases

	Histological type	N (%)
**Benign**	Endometriosis	17 (24.2)
	Inflammatory	5(7.1)
	*Tubo-ovarian abscess*	*1*
	*Hydrosalpinx*	*2*
	*Salpingo-oophoritis*	*2*
	Cystadenoma / cystadenofibroma	20 (28.5)
	Brenner	1 (1.4)
	Mature cystic teratoma	3 (4.2)
	Struma ovarii	3 (4.2)
	Haemorrhagic cyst	9(12.8)
	Simple cyst	8 (11.4)
	Leiomyoma	4(5.7)
	**Total**	70 (100)
**Malignant**	Borderline Ovarian Malignancy	8 (26.6)
	Serous	*6*
	Mucinous	*2*
	Epithelial Ovarian Malignancy	11 (36.6)
	Serous	*8*
	Mucinous	*2*
	Transitional	*1*
	Non-Epithelial Ovarian Malignancy	6 (20.0)
	Granulosa cell tumor	*4*
	Sertoli cell tumor	*1*
	Yolk sac tumor	*1*
	Metastatic ovarian malignancy	4(13.3)
	Small bowel	*1*
	Appendix	*1*
	Stomach	*1*
	Endometrium	*1*
	Non ovarian	1 (3.3)
	Endometrial stromal sarcoma of uterus	*1*
	**Total**	30 (100)

The median CA125, HE4, RMI and ROMA values were found to be significantly different between benign and malignant tumours (p< 0.0001 for each comparison) and are shown in table 2. The median values observed for CA125 in case of benign diseases were <35U/ml for cystadenoma/ cystadenofibroma, mature teratoma, struma ovariii and fibroids. The value of CA125 ≥35U/ml in case of benign diseases was observed for endometriosis, haemorrhagic cyst, inflammatory conditions, and simple cyst. In case of malignant cases median values above standard cut off were observed in each subcategory (table 2).

**Table 2:** Serum CA125, HE4, RMI and ROMA levels (median/range) and distribution according to histological types

		CA125^[1]^(U/m l)	HE4^[2]^(pM)	RMI^[3]^	ROMA^[¶]^ (%)	CA125	HE4	CA125 <35	CA125 <35	CA125 ≥35	CA125 ≥35
	N	Median (Range)	Median (Range)	Median (Range)	Median (Range)	≥35	≥70	HE4 <70	HE4 ≥70	HE4 <70	HE4≥70
**Benign Histology**											
Cystadenoma /cystadenofibroma	20	24.7 (10.0-190.9)	41.4 (30.0-159.3)	21.2 (0.0-423.0)	4.9 (2.4-59.3)	6	1	14	0	5	1
Haemorrhagic cyst	9	61.6 (7.0-1650.0)	42.3 (26.1-83.5)	23.8 (0.0-4950.0)	5.8 (1.8-25.0)	6	1	3	0	5	1
Benign cyst	9	35.4 (9.9-467.0)	49.6 (34.6-67.8)	0.0 (0.0-153.3)	7.7 (3.2-15.5)	5	0	4	0	5	0
Mature Teratoma	3	34.4 (33.8-40.0)	36.6 (28.6-49.3)	34.4 (0.0-120.0)	3.9 (2.2-7.6)	1	0	2	0	1	0
Struma ovarii	3	21.4 (17.2-1000.0)	60.9 (43.9-71.6)	64.2 (17.2-3000.0)	14.3 (5.7-16.0)	1	1	1	1	1	0
Endometriosis	17	145.6 (25.0-739.0)	47.5 (29.4-101.2)	103.9 (0.0-1701.0)	7.6 (2.4-34.1)	15	1	2	0	14	1
Inflammatory	5	49.7 (24.7-113.1)	63.7 (30.5-80.4)	100.8 (0.0-339.3)	13.1 (2.6-20.4)	3	1	1	1	3	0
Fibroid	4	25.3 (10.8-84.8)	46.0 (32.9-59.5)	76.0 (32.2-254.4)	6.6 (2.8-11.3)	1	0	3	0	1	0
**Malignant Histology**											
Borderline	8	118.0 (24.1-1365.0)	53.0 (15.0-35.0)	333.3 (0.0-4095.0)	10.2 (0.5-53.2)	7	3	0	1	5	2
Epithelial cancer	11	197.0 (19.8-3494.0)	151.5 (54.9-1059.0)	347.4 (0.0-10482.0)	55.3 (10.3-99.2)	10	9	0	1	2	8
Nonepithelial Cancer	6	208.75 (8.0-1010.0)	64.4 (45.6-80.0)	490.0 (0.0-3030.0)	14.7 (5.9-86.1)	4	3	2	0	1	3
Metastatic ovarian cancer	4	106.5 (39.8-420.0)	73.3 (44.6-27.0)	319.5 (119.3-1260.0)	19.6 (6.1-98.2)	4	2	0	0	2	2
Non ovarian cancer	1	57.0	41.9	171.00	5.5	0	0	0	0	1	0

**Figure 1:**
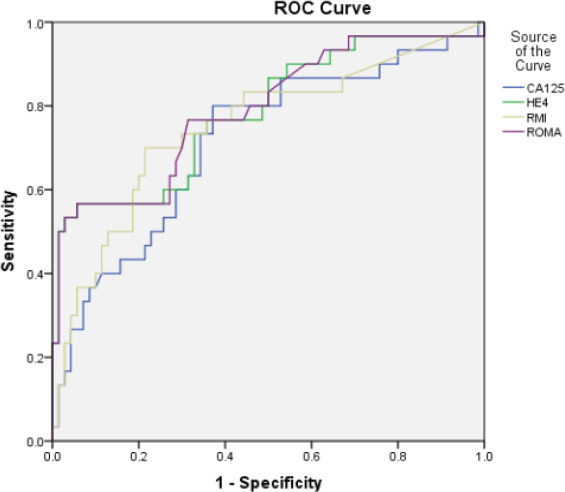
ROC curve for all ovarian malignancies' vs benign

**Figure 2:**
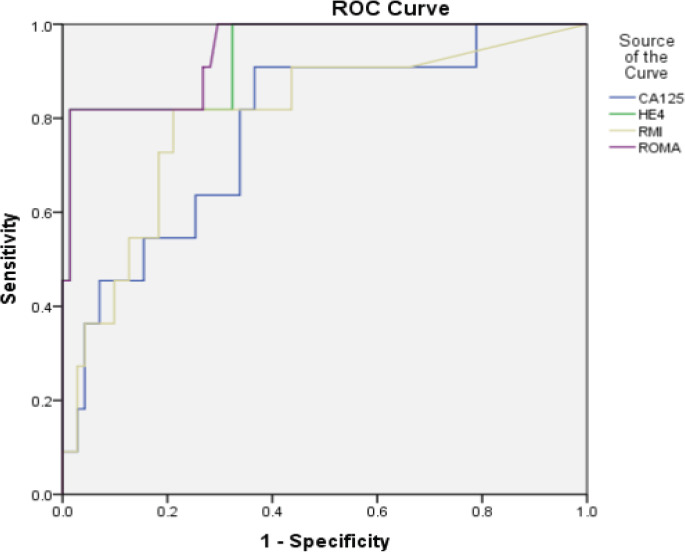
ROC curve for epithelial ovarian malignancies vs benign

**Figure 3:**
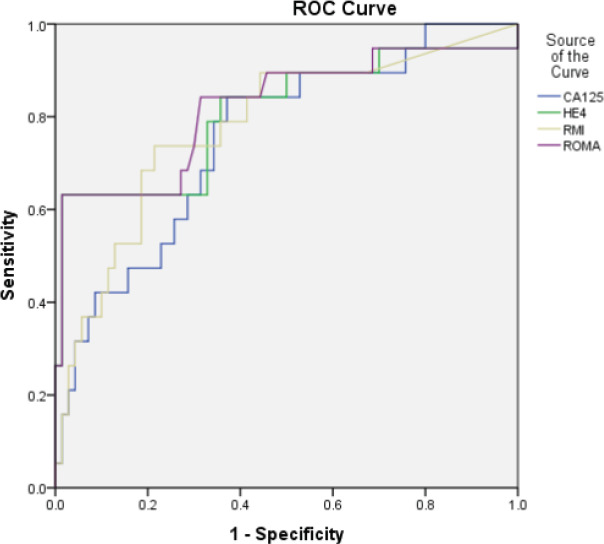
ROC curve for epithelial ovarian malignancies including borderline tumours compared with benign

**Figure 4:**
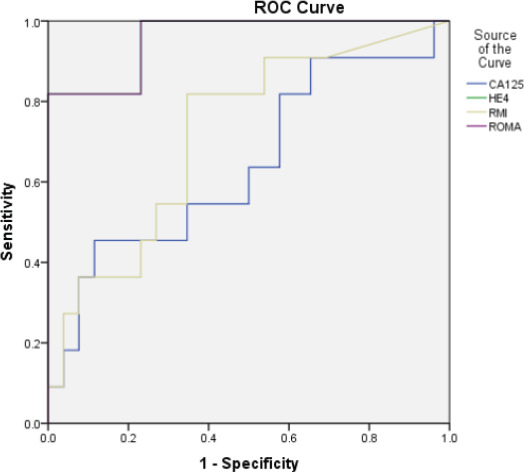
ROC Curve for Epithelial Ovarian Malignancies Compared with Endometriosis

Median values of HE4 were below cut off for each category of benign diseases. However, among malignant histology, median HE4 levels above cut off were observed only in case of epithelial ovarian cancers (EOC) and metastasis (table 2). Number of cases with CA125 and HE4 values above and below cut off in each of the category are shown in table 2. HE4 was able to identify two cases of malignancy missed by CA125, one case of borderline mucinous tumour and another case of serous epithelial ovarian cancer while HE4 was found to be falsely negative in 11 cases of malignancy that had CA125 positive. The cases included 2 cases of EOC (1 serous, 1 mucinous), 1 case of nonepithelial ovarian cancer (NEOC) (granulosa cell tumour), 2 cases of secondary ovarian metastatis from gastrointestinal tract, 1 case of non-ovarian malignancy (endometrial stromal sarcoma of the uterus) and 5 cases of borderline tumours (4 serous, 1 mucinous) In case of RMI, the observed median values found below the cut off in each category of benign diseases and above the cut off for each category in malignant diseases except in case of non-ovarian cancer. In case of ROMA, the observed median values in case of benign diseases were found below the cut off in case 123 of cystadenoma/ cystadenofibroma, mature teratoma, fibroids, endometriosis, haemorrhagic cyst, simple cyst. Median values above the cut off for benign diseases were observed in cases of struma ovarii and inflammatory pathology. Among malignant histology, median values above the cut off were observed in case of epithelial ovarian cancers, non-epithelial cancer and secondary ovarian cancers. Median values were found below the cut off in case of borderline histology and non-ovarian cancer. Out of all malignant cases CA125 was found positive in 86.6% while HE4 was found positive only in 56.6% of the cases. Both markers were found negative in 2 cases of malignancy, one case of granulosa cell tumour and one case of sertoli cell tumour. The sensitivity of CA125 to detect epithelial ovarian malignancy and malignancy in general was higher than HE4 but the PPV was lower. The specificity of HE4 was almost double as compared to CA125. The PPV of HE4 was found to be much higher but had comparable NPV. The sensitivity of HE4 was better in detecting EOC as compared to EOC plus borderline and malignancy in general. ROC curves were generated for all malignancies, EOC including borderline and EOC excluding borderline tumours. Area under curve (AUC) were obtained and 95% confidence interval limits for AUC were calculated. Pairwise comparison of the ROC-AUC for the 4 markers was done using Medcalc software for any significant difference. When Receiver operator characteristic (ROC) curve was drawn for all the malignant cases (Figure 1), malignant epithelial malignancies (Figure 2) and malignant epithelial malignancies including borderline tumours (Figure 3) against benign lesions, the AUC obtained was highest for ROMA (0.791) followed by HE4 (0.784), RMI (0.750) and CA125 (0.715). When ROC curve was drawn for EOC compared with endometriosis (Figure 4), as expected, HE4 and ROMA were found to be significantly able to differentiate between the two conditions (p value 0.001).

## Discussion

CA125 is the most widely used biomarker for ovarian malignancy. But despite having high sensitivity it is known to have high false positive rates. Preoperative CA125 assay is more valuable for postmenopausal women as compared to premenopausal women. American College of Obstetricians and Gynaecologists had previously recommended arbitrary cut-off > 200 U/L for referral of premenopausal women, but it was not supported by research evidence (14). High sensitivity and specificity obtained through pattern recognition ultrasound features is true only for USG done by experienced clinicians. Lactate Dehydrogenase (LDH), Alpha fetoprotein and beta human chorionic gonadotropin (HCG) recommended to be measured in all women less than 40 years of age but it is not done routinely due to low diagnostic performance and increase in cost (15). The inability of CA125 and existing other markers to accurately diagnose ovarian malignancies has led to a search for new marker with more accuracy. Several publications have shown HE4 to be a more specific marker than CA125. Test kits for HE4 are US FDA approved and there are algorithms designed including HE4 for malignancy risk prediction. But still there are no existing guidelines for its use in pre-menopausal age. The low prevalence of malignancy and higher prevalence of endometriosis and other benign diseases in premenopausal women and the need for ovarian function conservation requires a marker which is more specific and sensitive.

The higher percentage of malignancy (30%) in our study was due to selection bias as these cases had pelvic masses and were selected for surgery. HE4 correctly classified all benign lesions with resultant higher specificity. CA125 was also below cut off in all benign cases except one case in which patient had ascites along with a fibroid. In our study, within the benign histology group, CA125 was found to be significantly elevated only in case of endometriosis (145.6U/ml) which was in concordance with studies done by Anastasi et al and others (16-18). Though the median values for haemorrhagic cysts, simple cysts and inflammatory conditions were above the cut off for CA125, they were not significantly elevated (table 2). For HE4, RMI and ROMA none of the category within the benign group had median levels above standard cut off values. The percentage of false positive cases for CA125 and HE4 were 54.2% and 7% respectively. Our findings correlated with those described in the study by Moore et al (18) in which CA125 levels were more often (29%) elevated compared to HE4 levels (8%) among benign ovarian tumours. In the malignant group, CA125 was found to be positive in 86.6% of cases compared to 56.6% in case of HE4 in our study. HE4 was found to be significantly lower in borderline cases as compared to other malignancies (table 2). The finding was consistently seen in various previous studies (7, 19, 20). But most of the studies included borderline tumors in the low-risk group and reported higher efficacy for HE4 compared to CA125. The studies which included borderline in the high-risk group, all concluded superior sensitivity of CA125 over HE4 (10, 17). The sensitivity and specificity calculations were affected by the percentage of endometriosis, NEOC, secondary ovarian tumours and borderline tumours in the study group, differences in cut off values for HE4 taken and classification of borderline tumours as low risk or high risk. We compared the whole group and at standard cut off values found highest sensitivity for CA125 (86.6%) compared to HE4 (56.6%) and highest specificity for HE4 (92.8%) compared to CA125 (45.7%) (table 3).

**Table 3:** Sensitivity and specificity for CA125, HE4, RMI AND ROMA at standard cut off values

	Sensitivity (%)	Specificity (%)	Positive Predictive Value	Negative Predictive Value
**All malignant Cases (n=30)**				
CA125^[4]^	86.6	45.7	40.6	88.8
HE4^[§]^	56.6	92.8	77.2	83.3
CA125 AND HE4	50.0	95.7	83.3	81.7
RMI^[€]^	60.0	81.4	58.0	82.6
ROMA^[¶]^	56.6	84.2	60.7	81.9
**Epithelial ovarian cancer (n=11)**				
CA125	90.9	45.71	20.8	96.96
HE4	81.8	92.8	64.2	97.0
CA125 AND HE4	72.7	95.7	72.7	95.7
RMI	72.7	81.4	38.0	95
ROMA	81.8	84.2	69.2	96.7
**Borderline Tumours and Epithelial Ovarian Cancer (n=19)**				
CA125	89.4	45.71	30.9	94.11
HE4	63.15	92.8	70.5	90.2
CA125 AND HE4	52.6	95.7	76.9	88.1
RMI	68.4	81.4	50.0	90.4
ROMA	63.1	84.2	52.1	89.3

The sensitivity of CA125 was found be higher than HE4 for malignancies in general and for EOC with and without including borderline tumours. The sensitivity of HE4 was found to be highest when only EOC were compared, reduced when borderline was included and was least for malignancy in general. This proves that HE4 is a marker specific for epithelial ovarian cancers. ROC AUC observed in our study were highest for ROMA. No statistical difference was found for the 4 markers when comparing malignancies in general and when EOC were compared with or without including borderline tumours. Statistically significant difference was obtained only when endometriosis was compared with malignant epithelial ovarian cancers in case of HE4 and ROMA.

## Conclusion

HE4 is not superior to CA125 for diagnosing ovarian cancer in premenopausal women. HE4 is more specific for epithelial ovarian cancer and it cannot be used as primary marker in general population. In suspicious adnexal masses with raised CA125 and lower levels of HE4 can reliably exclude epithelial ovarian malignancy. Study concludes that HE4 can be used in series or as part of diagnostic algorithm (ROMA) along with CA125 to get higher diagnostic accuracy. ROMA is more reliable than HE4, CA125 or RMI in diagnosing ovarian malignancy in premenopausal women. Much larger study is required before HE4 and ROMA could be suggested as routine investigations for evaluation of suspected adnexal masses.
